# Stem cell therapy: a novel treatment option for cerebral malaria?

**DOI:** 10.1186/s13287-015-0138-6

**Published:** 2015-08-08

**Authors:** Wei Wang, Hui Qian, Jun Cao

**Affiliations:** Jiangsu Institute of Parasitic Diseases; Key Laboratory on Technology for Parasitic Disease Prevention and Control, Ministry of Health; Jiangsu Provincial Key Laboratory of Molecular Biology of Parasites, 117 Yangxiang, Meiyuan, Wuxi City, Jiangsu Province 214064 China; School of Medicine, Jiangsu University, 301 Xuefu Road, Zhenjiang City, Jiangsu Province 212013 China

## Abstract

Cerebral malaria, a severe form of the disease, is one of the most severe complications of infection with *Plasmodium* parasites and a leading cause of malaria mortality. Currently available antimalarial therapy has proven insufficient to prevent neurological complications and death in all cases of cerebral malaria. Souza and colleagues observed that transplantation of bone marrow-derived mesenchymal stromal cells (BM-MSCs) increased survival, reduced parasitemia, decreased malaria pigment accumulation in the spleen, liver and kidney, elevated Kupffer cell count in liver, alleviated renal injury and lung inflammation, and improved lung mechanics in an experimental mouse model of cerebral malaria. Although plenty of challenges lie ahead, their findings show the promise of BM-MSC therapy for the treatment of cerebral malaria.

As one of the most severe complications of infection with *Plasmodium* parasites, cerebral malaria may cause neurological disorders that manifest as severe headache, drowsiness, confusion, coma, and convulsion, and affect other vital organs such as lung, kidney, heart, spleen and liver. Worldwide, cerebral malaria is a leading cause of malaria mortality, responsible for almost 20 % of adult deaths and 15 % of childhood deaths. To date, there is no effective and safe treatment available for cerebral malaria, notably for use in children, who represent the majority of cases. Even patients that are given standard antimalarial therapy at an early phase still face a high risk of dying despite clearance of the parasite, while approximately 25 % of survivors may develop neurological complications and cognitive impairment. In a recent issue of *Stem Cell Research & Therapy*, Souza and colleagues [1] observed that administration of bone marrow-derived mesenchymal stromal cells (BM-MSCs) increased survival and reduced parasitemia and malaria pigment deposition in the spleen, liver, kidney and lung in an experimental mouse model of cerebral malaria. Their findings may provide a new option for the treatment of cerebral malaria.

To date, stem cell therapy has shown satisfactory outcomes in the treatment of a wide variety of human diseases, mainly tissue injury and immune disorders [2]. With the hope of conquering parasitic diseases, stem cell therapy has been introduced for parasitic infections since the 1990s [3], with the most successful attempt seen in Chagas disease [4], an emerging global health threat caused by the parasite *Trypanosoma cruzi* that may cause fatal heart diseases [5]. The value of stem cell therapy for malaria was firstly documented in 1991, when Japanese scientists found that multipotent hemopoietic stem cells contributed to host defense against *Plasmodium berghei* infection, and increased the survival of infected mice [6]. Subsequently, transplantation of myeloid cells produced by interleukin 7 receptor-alpha (IL-7Rα)^+^ c-Kit^hi^ progenitors, which were isolated from mice experimentally infected with *P. chabaudi*, was found to result in clearance of infected erythrocytes in infected mice, and promote recovery from the infection [7]. The advantages of high self-renewal ability, multilineage differentiation, wide presence, easy isolation and rapid expansion facilitate the ‘gold rush’ to use MSCs, and the number of clinical trials on MSCs has been rising since 2004 [8]. As a consequence, the *in vivo* efficacy of MSC treatment against malaria was evaluated in a mouse model of *P. berghei* infection; administration of MSCs was found to confer host resistance against malaria through increasing IL-12 production, and suppressing IL-10 and regulatory T cell production [9]. Indeed, these findings demonstrate the promise of MSCs in the treatment of malaria; however, no data are available on the effect of MSC therapy on organs affected due to cerebral malaria.

The first insight from this study is that intravenous injection of BM-MSCs results in reduced mortality and parasitemia, decreased malaria pigment accumulation in the spleen, liver and kidney, elevated Kupffer cell count in liver, alleviated renal injury and lung inflammation, and improved lung mechanics in mouse cerebral malaria models experimentally infected with *P. berghei*. Surprisingly, however, these alterations seem not be associated with changes in the expression of pro-inflammatory cytokines, including interferon-gamma, tumor necrosis factor alpha and chemokine (C-X-C motif) ligand 1 (CXCL1) as detected using enzyme-linked immunosorbent assays 5 days post-infection, but rather to reduced peripheral parasitemia. As stated in the discussion, the duration of observation seems relatively short. In the future, the time-course effect of MSC therapy on *Plasmodium* infection should be evaluated over an extended period of time. Since multiple cell tracking strategies have been developed to track the migration, distribution and target tissues of intravenously injected stem cells [10], tracking of labeled MSCs given to the animal models is required in future studies.

Second, this study demonstrates that administration of BM-MSCs results in elevated counts of astrocytes and oligodendrocytes in *P. berghei*-infected mice, which implicates tissue repair. Interestingly, assessment of brain injury with a five-point, semi-quantitative, severity-based scoring system revealed no significant difference in tissue inflammation or severity of histoarchitectural damage between the treated and untreated infected mice, indicating that BM-MSC therapy does not affect the inflammatory process of the brain tissues. In addition, BM-MSC therapy was found to mitigate renal injury caused by *P. berghei*, while it did not improve renal function, as reflected by no significant difference in serum creatinine, blood urea nitrogen, blood urea nitrogen/serum creatinine ratio or urinary creatinine between the treated and untreated groups. Further studies to investigate the exact mechanisms underlying these differences seem justified.

However, the use of rodent malaria parasite for modeling experimental cerebral malaria remains controversial, since the major difference between human cerebral malaria and the *P. berghei* ANKA models is thought to be the extent of parasite sequestration in the brain [11]. Therefore, more evidence should be obtained from nonhuman primate models prior to the consideration of clinical trials of this new treatment option. Additionally, the issue of safety related to BM-MSC therapy is not addressed in this study.

## Conclusion

Souza and colleagues [1] evaluate, for the first time, the effects of BM-MSC therapy in multiple organ dysfunction during experimental cerebral malaria, and demonstrate that administration of BM-MSCs results in reduced mortality and parasitemia. Although plenty of challenges lie ahead, the results of this study validate the promise of BM-MSC therapy in the treatment of cerebral malaria. The ultimate goal of any intervention is to improve clinical outcome and prognosis. Hence, although the study by Souza and colleagues is a major advance towards the treatment of cerebral malaria, we still need to devise optimal strategies to improve the control of encephalopathy and other complications of cerebral malaria.

## Abbreviations

BM-MSC: Bone marrow-derived mesenchymal stromal cell
IL: Interleukin
MSC: Mesenchymal stromal cell
